# On the Residual Stresses and Fracture Toughness of Glass/Carbon Epoxy Composites

**DOI:** 10.3390/ma15207135

**Published:** 2022-10-13

**Authors:** M. A. Umarfarooq, P. S. Shivakumar Gouda, N. R. Banapurmath, M. I. Kittur, Tabrej Khan, Irfan Anjum Badruddin, Sarfaraz Kamangar, Mohamed Hussien

**Affiliations:** 1 Center of Excellence in Material Science, School of Mechanical Engineering, KLE Technological University, Hubballi 580031, India; 2 Department of Mechanical Engineering, SDM College of Engineering & Technology, Dharwad 580002, India; 3 School of Mechanical Engineering, KLE Technological University, Hubballi 580031, India; 4 Centre of Advanced Materials, Faculty of Engineering, Universiti Malaya, Kuala Lumpur 50603, Malaysia; 5 Department of Mechanical Engineering, Faculty of Engineering, Universiti Malaya, Kuala Lumpur 50603, Malaysia; 6 Department of Engineering Management, College of Engineering, Prince Sultan University, Riyadh 11586, Saudi Arabia; 7 Mechanical Engineering Department, College of Engineering, King Khalid University, Abha 61421, Saudi Arabia; 8 Department of Chemistry, Faculty of Science, King Khalid University, P.O. Box 9004, Abha 61413, Saudi Arabia; 9 Pesticide Formulation Department, Central Agricultural Pesticide Laboratory Agricultural Research Center, Dokki, Giza 12618, Egypt

**Keywords:** interlaminar fracture toughness, hybrid composites, residual stresses, slitting method, SEM

## Abstract

The resistance to delamination in polymer composite depends on their constituents, manufacturing process, environmental factors, specimen geometry, and loading conditions. The manufacturing of laminated composites is usually carried out at an elevated temperature, which induces thermal stresses in composites mainly due to a mismatch in the coefficient of thermal expansion (CTE) of fiber and matrix. This work aims to investigate the effect of these process-induced stresses on mode-I interlaminar fracture toughness (G_I_) of Glass-Carbon-Epoxy (GCE) and Glass-Epoxy (GE) composites. These composites are prepared using a manual layup technique and cured under room temperature, followed by post-curing using different curing conditions. Double cantilever beam (DCB) specimens were used to determine G_I_ experimentally. The slitting technique was used to estimate residual stresses (longitudinal and transverse direction of crack growth) inherited in cured composites and the impact of these stresses on G_I_ was investigated. Delaminated surfaces of composites were examined using a scanning electron microscopy (SEM) to investigate the effect of post-curing on the mode-I failure mechanism. It was found that G_I_ of both GE and GEC composites are sensitive to the state of residual stress in the laminas. The increase in the G_I_ of laminates can also be attributed to an increase in matrix deformation and fiber–matrix interfacial bond with the increase in post-curing temperature.

## 1. Introduction

The most important characteristic of the composites is their flexibility to tailor the desired properties in the application environment. The preferred properties in a laminated polymer composite can be achieved by using different fibers and orientations, matrix materials, manufacturing processes, and curing conditions. Polymer composites reinforced with multiple fibers, known as hybrid composites, have provided the designers with an option to exploit the superior properties of fibers by mitigating the weakness of individual fibers. Hybridization of fibers in composites has the advantage of replacing the expensive fibers with cheaper ones, thereby reducing the overall cost of the composites without compromising on their functional requirements. Hybrid polymer composites are anisotropic and due to the differences in CTE between the fibers and matrix, thermal stresses develop in the composite during their processing. The nature of residual stresses developed in the hybrid composite is different from those in the composite reinforced with a single type of fiber [1,2,3,4].

Ply delamination is a major cause of failure in the laminated composite. Delamination in a composite affects the strength, stiffness, and structural integrity of the composites. The resistance to delamination in a material which is defined by the fracture toughness is an important property of composites. Therefore, the factors which affect fracture toughness need to be thoroughly investigated. The stresses induced during the curing stage are found to have a considerable effect on the fracture toughness of the composite. Knowledge of the magnitude of process-induced stresses and their behavior is of utmost importance. Therefore, to avoid non-conservative designs their effect can be incorporated during the design stage of the composite [5,6].

According to Nairn [7], the fracture toughness estimated using DCB specimen by ignoring residual stresses is apparent rather than true toughness. By considering residual stresses into account, the corrected beam theory was used to predict toughness. The error in the critical energy release rate (ERR) of DCB specimens on different Graphite/Epoxy laminates caused by ignoring residual stresses ranged from −54.85% to +76.36%. Laik et al. [8] investigated the effect of residual stresses on the G_I_ and shear strength of CE composites prepared by vacuum-aided resin transfer molding. The residual stress profile in CE laminates post-cured at different temperatures was determined using FE analysis. Process-induced stresses were found to have a positive effect on ERR and a negative effect on in-plane shear strength. Gillespie et al. [9] used a numerical model to investigate the effect of different parameters such as laminas stacking, thickness of composite, rate of cooling, and thermal conductivity on the formation of residual stresses in thermoplastic composites. A higher cure-induced stress resulted in a 35% deterioration in critical ERR. In unidirectional (UD) CE laminates, the different cooling rates had negligible effect on G_I_ and residual stresses along fiber directions developed had minimal variations. It was concluded that the residual stresses along the direction of crack propagation did not affect the G_I_ of the FRP composites. Nelson et al. [10] conducted a series of experiments using DCB specimens of CE, GE, and GCE composites at 71 °C, 25 °C, and −54 °C. However, no noticeable change in fracture toughness due to the effect of temperature range was observed in symmetric GE and CE composites. Nevertheless, fracture toughness of asymmetric GCE composite showed a significant dependence on temperature. These results were used to aid the FE simulation to understand the improvement in presence of residual stresses on G_I_ of asymmetrical GCE DCB composite laminates. Oliver et al. [11] studied the influence of cure stresses on the mechanical properties of CE composite manufactured using an autoclave. The profile of cure stresses in composites was changed using different curing conditions and assessed by measuring out-of-plane distortion in composites. The results showed that residual stresses had a beneficial effect on the transverse and longitudinal tensile strength of composite laminates. Robinson et al. [12] investigated the impact of residual stresses on the G_I_ of different cross-ply [0/90] DCB specimens with a delamination crack interface at 0°//0°. The transverse tensile residual stresses resulted in the reduction of G_I_ and the bend-twist coupling across the arms of the DCB specimen caused significant alterations in the opening mode fracture toughness. Furthermore, the authors [13] investigated the effect of cure-induced stresses on G_I_ in CE composite laminates. The compressive residual stresses were found to have a positive impact on the G_I_ of CE laminates. Nevertheless, no systematic investigation has been reported to understand the influence of process-induced stresses on G_I_ in hybrid fiber-reinforced polymer composites. The present work aims to study the influence of process-induced residual stresses on mode-I interlaminar fracture toughness of GE and hybrid GCE composite laminates.

## 2. Experimental Details

### 2.1. Materials

UD Carbon (*Toray T300*) and UD Glass fiber (*Interglas 92145/Finish FK 144*), supplied by *Mark-Tech Composites Pvt. Ltd.* Bengaluru, India were used as reinforcements for this study. The matrix used was composed of Epoxy (*Araldite LY-556*) cured by Catalyst (*Aradur 917*) added in a volumetric ratio of 9:1.

### 2.2. Fabrication of Composite Laminates

**Figure 1:**
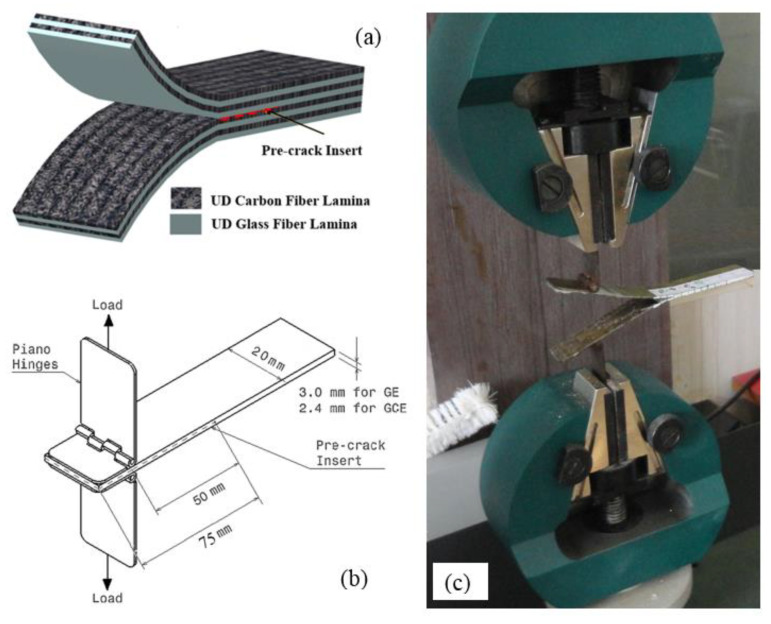
Schematic illustrations depicting (**a**) Lamina layup of GCE laminates. (**b**) Specification of DCB specimen. (**c**) Mode-I fracture test of GCE specimen.

GE [0]_12_ and GCE [0^Glass^/0^Carbon^]_4_ composite laminates were prepared by manual layup technique. Initially, the samples were cured at room temperature. DCB Laminates were prepared by embedding a polyethylene sheet of 20 µm thickness which acts as a pre-crack interface and yields symmetric beams of equal thickness. The arrangement of laminas in hybrid GCE specimen is shown in Figure 1a. Specimens for mode-I fracture testing were cut following ASTM D 5528 [14]. Subsequently, the samples were post-cured at different temperatures.

#### Post-Curing of the Composite Laminates

The residual stresses in the composites were varied by post-curing at different temperatures. GE and GCE composite laminates were post-cured at 90 °C, 135 °C, and 180 °C for 6 h and cooled to room temperature with a cooling rate of 20 °C/min. The post-cured GCE and GE composite samples are coded and enlisted in Table 1 according to their respective post-curing conditions.

## 3. Mechanical Tests

### 3.1. Mode-I Fracture Test

Mode-I Fracture toughness of composite laminates was determined using DCB specimen following ASTM D 5528. Testing was carried out with a displacement control mode having a speed of 2 mm/min using *Tinius Olsen* UTM having a load cell capacity of 10 kN, with a least count of 0.01 N. Specifications of DCB specimen with initial delamination of 75 mm from one end are shown in Figure 1b. DCB specimens were loaded through piano hinges bonded using a strong adhesive (*Araldite*) to the top and bottom beams of the specimen as shown in Figure 1c. A paper scale was pasted on the top of the specimen and white paint was applied across the thickness to enhance the visualization of the crack tip. On the paper scale, vertical lines were marked starting from the end of the *Teflon* sheet in increments of 1 mm up to 5 mm. The remaining 45 mm was marked with vertical lines in increments of 5 mm. The commencement of the crack and its successive propagation were recorded by the *Sony* HD video recorder. The corresponding load at initiation and subsequent growth were documented in the load–displacement curve. G_I_ was evaluated using modified beam theory using the expression given in Equation (1).

(1) $$G_{I}=\frac{3P\delta }{2b(a+\left|\Delta \right|)}$$

where P is the applied load, δ is the load-point displacement, *b* is the width of the specimen, *a* is the length of delamination, and Δ is the correction factor to account for the rotation of the DCB arms. Δ is determined experimentally by drawing a least-squares plot from a graph of the cube root of compliance versus delamination length.

### 3.2. Residual Stress Determination Using Slitting Method

**Figure 2:**
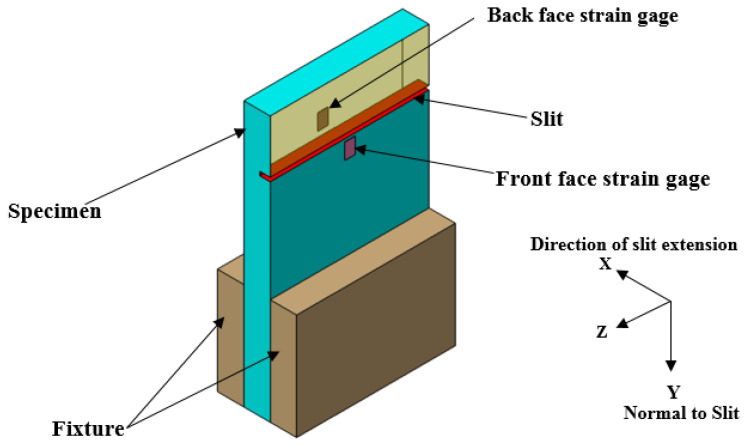
Details of Slitting Specimen.

Destructive methods of residual stress measurements are based on the principle of estimating stresses from deformations/strains, which are released when the stressed specimen is locally machined. The slitting method [15,16,17,18,19,20,21,22,23,24,25], also known as the crack compliance method, is one of the most commonly used destructive methods. It involves machining a narrow slit through a stressed sample and the released strains are measured using a strain gage bonded across the adjacent material. The basic principle of slitting can be described with a usual slitting specimen shown in Figure 2.

A slit is machined from the front face in the form of incremental cuts along the X-direction, the released strains due to each cut are recorded by strain gauges bonded to the material adjacent to the slit as shown in Figure 2. These recorded strains are used to calculate the residual stresses. The slitting method can measure residual stresses only perpendicular to the slit.

The relationship between relaxed strains and residual stresses to be determined is complex, as the location of the released strains and their measurement is in different region. The relationship between measured strains and the residual stresses is of integral form and is given by Equation (2) [13]:

(2) $$\varepsilon_{yy}\;\left(a_{i}\right)=\;\int_{0}^{a_{i}}C\;(x,a_{i})\;\sigma_{yy}\left(x\right)\;dx\;$$

where *ε_yy_* (*a_i_*) are the measured strains when the depth of slit is *a_i_*, $\sigma_{yy}$ are the residual stresses to be determined, and $C(x,a_{i})$ is the kernel function, which implies the measured strains when stress of unit value is applied at depth ‘*x*’ within a slit of depth ‘*a_i_*’ and is determined using finite element (FE) analysis.

To solve Equation (2), it is essential to assume the initial distribution of the unknown residual stresses. The residual stresses calculated depend on the function, which defines how the residual stresses are assumed to be varying in the material. For laminated composites, the stresses vary across each layer. Therefore, a stress formation technique known as the “pulse method” is chosen to approximate the stresses. In the pulse method, stresses are assumed to be uniform over each incremental cut and the residual stresses that are given by Equation (3).

(3) $$\sigma \left(x_{j}\right)=\sum_{j=1}^{n}\sigma_{j}U_{j}\left(x\right)$$

where ‘*σ_j_*’ implies the stress in ‘*j*_th_’ incremental cut when ‘the total number of incremental cuts is ‘*n*’. The pulse functions [U_j_(x)] are defined as

(4) $$U_{j}\left(x\right)=\{\begin{array}{c} 1\;\;\;\;\;\;\;\;\;\;a_{j-1}\leq x\leq a_{j}\\ 0\;\;\;\;\;\;\;\;\;\;x\langle a_{j-1},x\rangle a_{j} \end{array}$$

The recorded strains and the residual stresses for each incremental cut can be expressed in matrix form as:

(5) [C]{σ}={ε}

where [*C*] is the compliance coefficient matrix, {σ} is the vector of residual stress and {ε}—vector of recorded strain compliance coefficients were determined by simulating the experimental slitting process of the composite specimen using FE analysis. The simulation was carried out using ANSYS software with the experimentally determined elastic constants of GE and CE laminates* using the procedure detailed in the earlier reported works [13,18,20,21,22,23,24,25]. The specific element of compliance matrix C_ij_ corresponds to strains measured across the strain gauge when the applied residual stresses are equal to the unit load.

Note: The elastic constants of Carbon/Epoxy (CE) composite laminates; Glass/Epoxy (GE) composite laminates; and CTE of Epoxy, Carbon and Glass fibers are provided in the Supplementary File.

### 3.3. Experimental Details

#### 3.3.1. Sample Preparation for Bonding of Strain Gage

**Figure 3:**
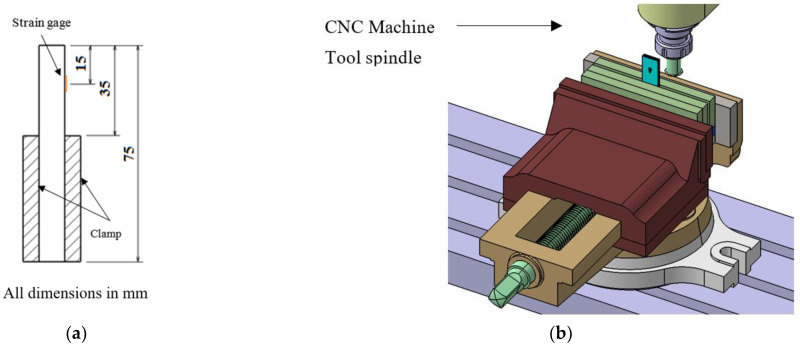

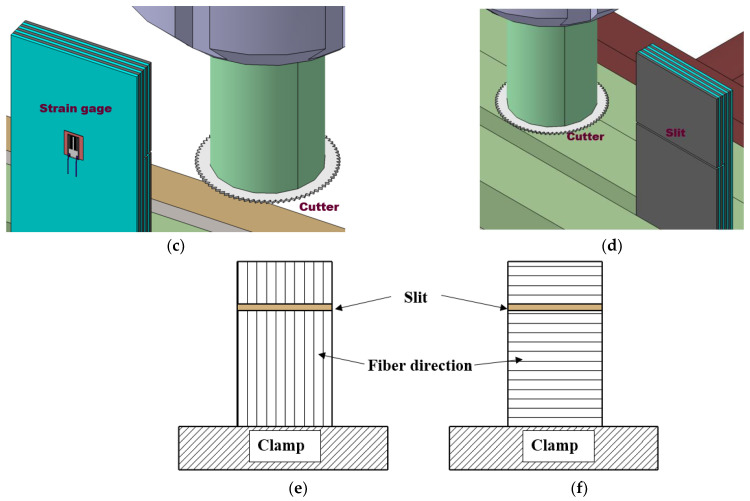
Schematic illustrations of (**a**) Slitting specimen with a bonded strain gage. (**b**) Experimental setup for the slitting experiment. (**c**) Relative position of strain gage and cutter (**d**) Relative position of cutter and slit. (**e**) Slitting specimen used for determination of longitudinal residual stress. (**f**) Slitting specimen used for determination of transverse residual stress.

The specimen dimensions used for the slitting process are given in Table 2. Before bonding the strain gage on composite laminate, the specimen was thoroughly washed with acetone to remove the dirt, grease, and oil. A strain gage of 1 mm gage length was bonded on the composite specimen at a distance of 15 mm from the top edge as shown in Figure 3a.

#### 3.3.2. Details of Slitting Experiment

Slitting of the composite laminates was conducted using a CNC machine (*BFW Surya VF 30 Vertical Machining Center*). The specimen was clamped at one end and a progressive slot was machined at the free end as shown in Figure 3b. The position of the cutter is carefully adjusted to machine the slot on the opposite face of the bonded strain gage. The slit was machined with a circular saw (HBM: Diameter: 20 mm and thickness: 0.3 mm) having rotational and translational speeds of 2000 rpm and 180 mm/min respectively. The depth of each incremental cut is equal to the thickness of each lamina of a composite laminate. Figure 3c,d show the relative position of the cutter, strain gage, and slit position during the slitting experiment. The slit was machined through the entire thickness of composite laminates. The strains released due to slitting were read by the digital strain indicator and recorded once they are stabilized. The slitting was carried out along and across the fiber directions to determine the residual stresses in longitudinal and transverse directions. The slitting specimen used to determine longitudinal and transverse residual stresses is shown in Figure 3e,f respectively.

## 4. Results and Discussions

### 4.1. Estimation of Residual Stresses

**Figure 4:**
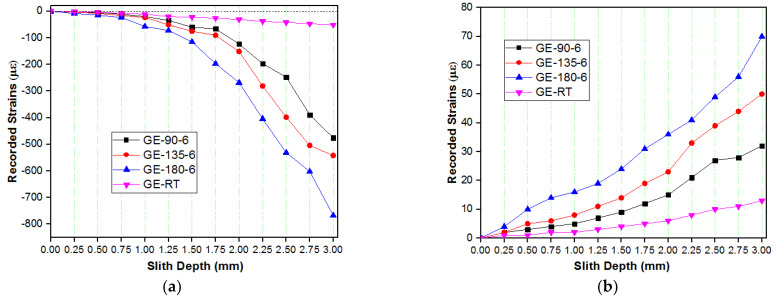

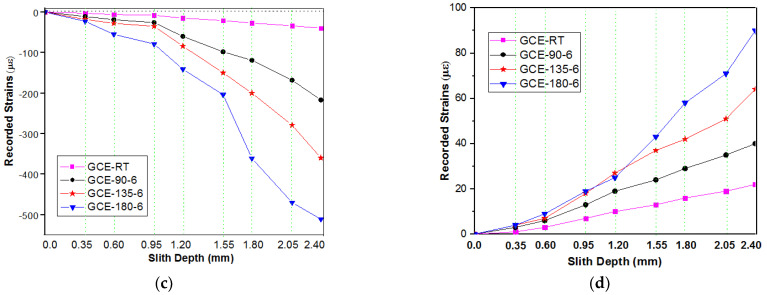
Strains recorded as a function of slit depth (**a**) Longitudinal Strains in each lamina from slitting of GE laminates. (**b**) Transverse strains in each lamina from slitting of GE laminates. (**c**) Longitudinal Strains in each lamina from slitting of GCE laminates. (**d**) Transverse strains in each lamina from slitting of GCE laminates.

The longitudinal and transverse strains recorded during the slitting of GE and GCE laminates in successive incremental cuts are shown in Figure 4. The strains increased with an increase in the depth of cut.

The process-induced stresses in the polymer composites depend on the temperature profile, the degree and duration of the temperature, and the rate of cooling. Moreover, the longitudinal and transverse residual stresses depend on constituents of composite laminate, ply orientation, fiber volume ratio, and processing conditions of the composite structure. The CTE of fibers is typically orthotropic and is usually lower than the polymer matrix material. The CTE of carbon fibers in the longitudinal direction is low or negative but higher along its transverse direction. This results in the development of stresses during cooling even in the UD material. The cure-induced strains in composite laminates increase as the post-curing temperature rises. The findings for unidirectional samples showed that stresses were only significant in the longitudinal direction and were negligible in the other two directions [24,26,27]. A similar phenomenon was observed by Sicot et al. [28] in UD Carbon Epoxy composite and Ghaedamini et al. [26] in UD Glass Epoxy composite. CTE of polymer matrix is higher than the fiber, typically an order of magnitude or more. For Carbon/Epoxy composites, the difference in CTE between the fiber (−1 × 10^−6^$\;\frac{m}{m℃}$) and matrix (60 × 10^−6^$\;\frac{m}{m℃}$) along longitudinal direction is high compared to that in transverse direction. Therefore, the stresses along longitudinal direction is higher compared to transverse direction. 

**Figure 5:**
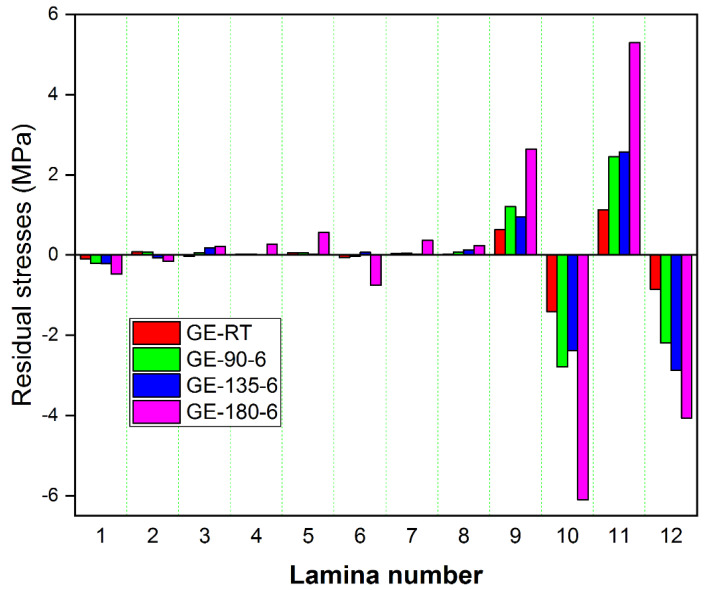
Transverse residual stress in each lamina determined from recorded transverse strains for all GE laminates.

**Figure 6:**
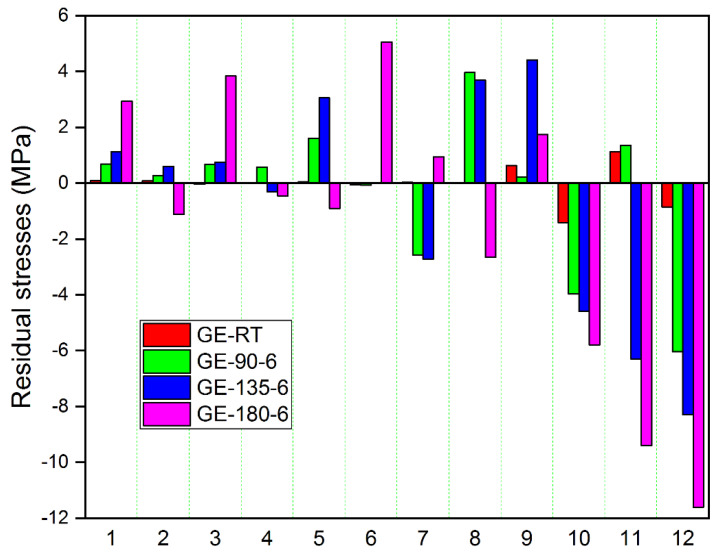
Longitudinal residual stress in each lamina determined from recorded longitudinal strains for all GE laminates.

**Figure 7:**
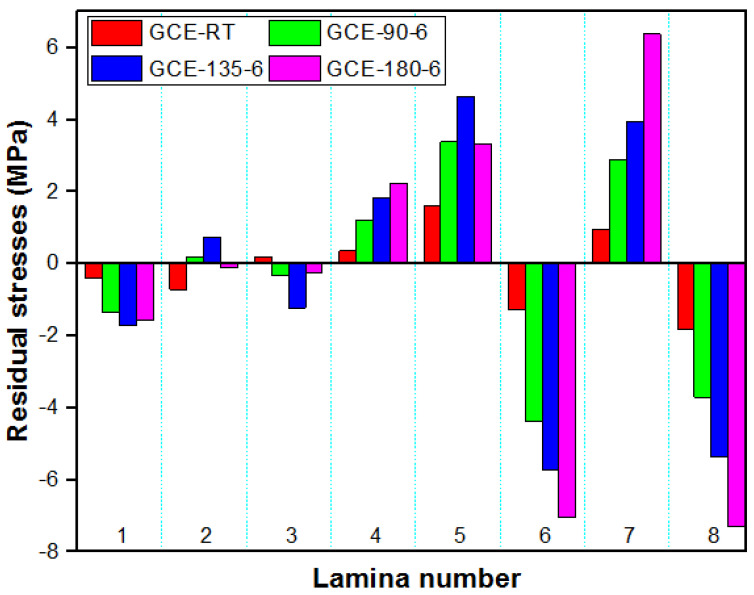
Transverse residual stress in each lamina determined from recorded transverse strains for all GCE laminates.

**Figure 8:**
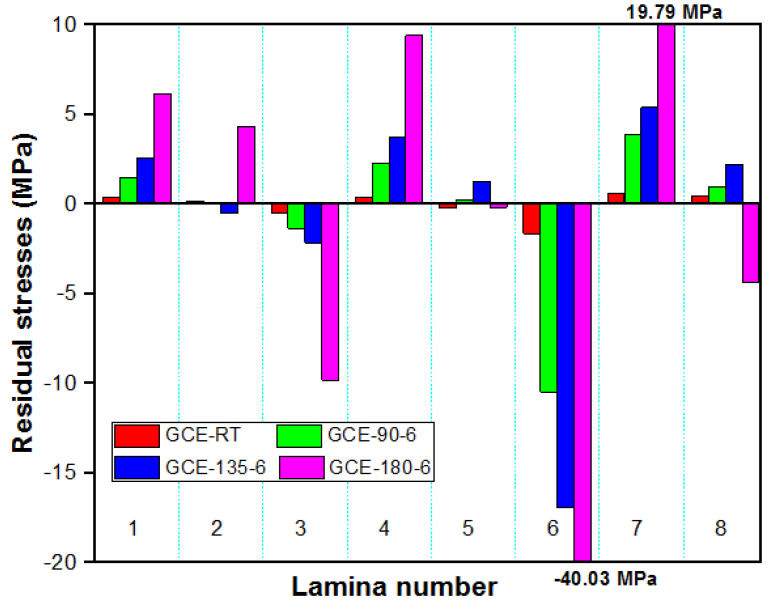
Longitudinal residual stress in each lamina determined from recorded longitudinal strains for all GCE laminates.

Specimens post-cured at 180 °C released more strains compared to other composites. The transverse and longitudinal strains in GE and GCE laminates gradually increased with post-curing temperatures. The laminates cured at RT released lower strains compared to other temperatures. The transverse and longitudinal residual stresses locked up in each lamina of GE laminates were determined from corresponding measured strains using the Pulse method, which are plotted in Figure 5 and Figure 6 respectively. Similarly, Figure 7 displays the variation of transverse residual stresses, and Figure 8 shows the variation of longitudinal residual stresses across each lamina of GCE. The strains recorded by the strain gauge for each incremental cut are a result of the overall interaction between the constituents of the composite laminates and processing conditions. Longitudinal residual stresses in GE and GCE composite laminates varied between −9.40 Mpa to +5.06 Mpa and −40.03 Mpa to +19.79 Mpa respectively. Transverse residual stresses in GE and GCE composite laminates varied between 6.10 Mpa to +5.29 Mpa and −7.31 Mpa to +6.38 Mpa respectively. Post-curing of the composite laminates initiated an increase in cure characteristics, which further increased the level of residual stresses in composite laminates. The summation of transverse residual stresses in GE and GCE laminates is reported in Table 3.

### 4.2. Mode-I Test Results

**Figure 9:**
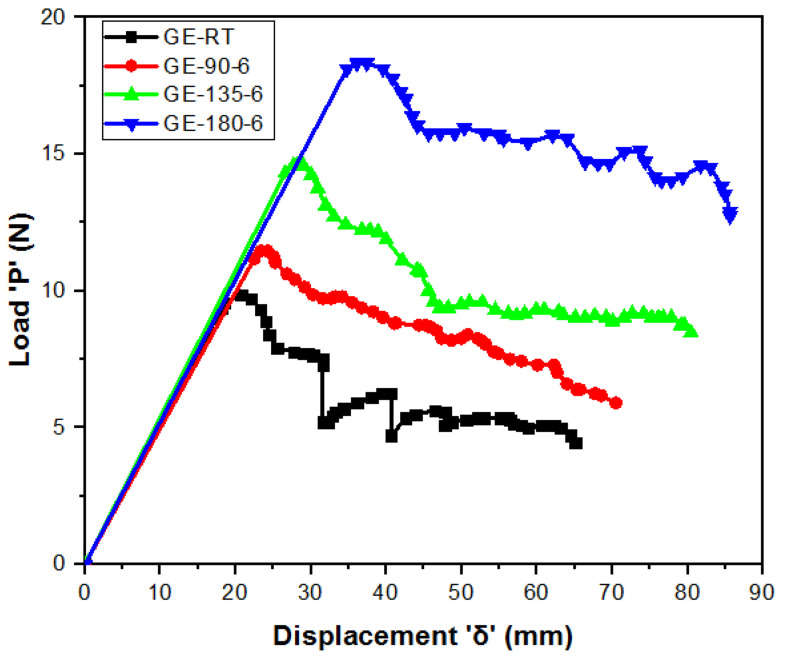
Load (P) versus Displacement (δ) plots from DCB tests of GE laminates.

**Figure 10:**
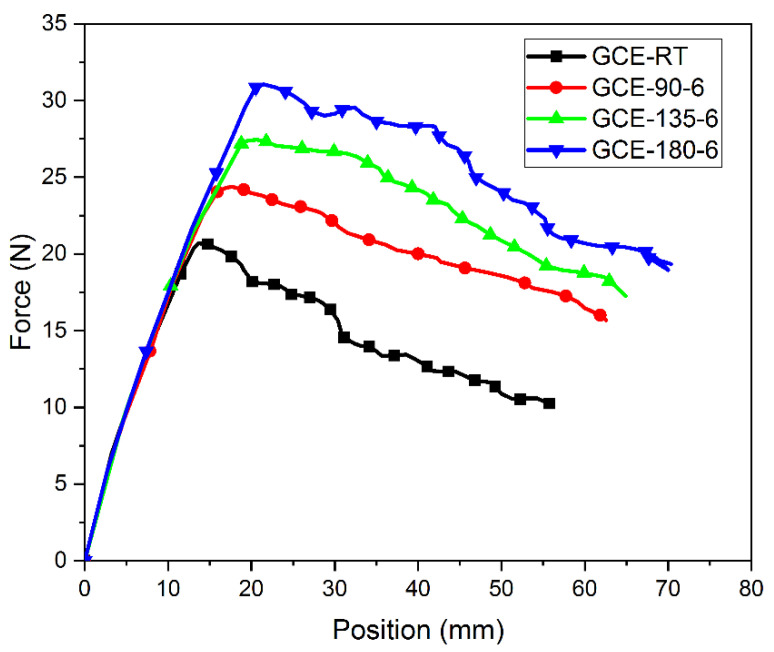
Load (P) versus Displacement (δ) plots from DCB tests of GCE laminates.

**Figure 11:**
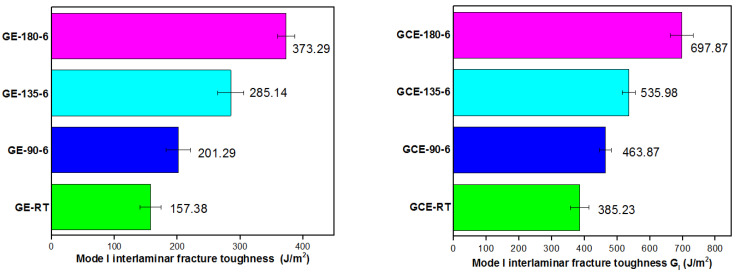
Mode-I interlaminar fracture toughness G_I_ of GE laminates (Left) and GCE laminates (Right).

Experimentally obtained load–displacement plots for all post-cured GE and GCE laminates are shown in Figure 9 and Figure 10, respectively. Mode-I interlaminar fracture toughness (G_I_) for GE and GCE laminates is plotted in Figure 11. Five samples from each post-cured condition were tested.

From Figure 9 and Figure 10, it can be observed that the slopes of GE *P-δ* and GCE *P-δ* curves are linear until they reach the maximum load point. Both the GE and GCE *P-δ* plots show no noticeable load drops. The crack growth in GE (except GE RT) and GCE laminates is stable and no ‘Stick-Slip’ behavior is observed when compared to CE *P-δ* curves [13]. From Figure 9 and Figure 10, it is noted that higher loads for initiation of crack are required in post-cured hybrid GCE laminates when compared to GE laminates. To investigate the influence of process-induced stresses on *G_I_* in GE and GCE laminates, the transverse residual stresses in the laminas tabulated in Table 3 are considered.

### 4.3. Effects of Residual Stresses on Interlaminar Fracture Toughness

Residual stresses in the laminas can be either tensile or compressive, based on their convention. They can contribute to the energy release rate either by doing additional work or by liberating strain energy as the crack propagates. Compressive residual stresses in laminas can add up to the energy release rate by doing additional work whereas tensile residual stresses contribute to the energy release rate by releasing the strain energy [7,8,29]. A gradual increase in transverse tensile residual stress in multi-directional Carbon-Epoxy composite with delamination plane at 0°//0° results in a decrease in *G_I_* [12]. The state of residual stress in the fiber direction (crack growth direction) was found to have a negligible effect on *G_I_* [9].

#### Influence of Residual Stresses on G_I_ in GE and GCE Composite Laminates

From Table 3, it can be observed that *G_I_* for GE-RT is 157.38 J/m^2^ with process-induced residual stress being −0.50 MPa. For the composite laminates post-cured at 90 °C and 135 °C, the process-induced residual stresses are −1.27 MPa and −1.62 MPa respectively with corresponding *G_I_* being 201.29 J/m^2^ and 285.14 J/m^2^ respectively. GE-180-6 laminates exhibit *G_I_* of 373.29 J/m^2^ with stresses induced being −1.98 MPa. Similarly in hybrid GCE laminates, as the transverse compressive residual stress in GCE laminates increase there has been a gradual rise in mode-I interlaminar fracture toughness. GCE-180-6 composites exhibit higher *G_I_* compared to other GCE composites and corresponding induced residual stresses were −4.32 MPa which is higher than other GCE composites. An increase in *G_I_* of GE and GCE laminates is observed with a gradual increase in the magnitude of residual stresses in composite laminates. As the crack propagates, compressive residual stresses contribute to the energy release rate in the form of additional laminate arms displacement [7,8,18,29].

## 5. Scanning Electron Microscopic (SEM) Analysis

To understand the effect of post-curing on the fracture mechanism of GE and GCE laminates under mode-I, fractured delaminated surfaces 10 mm ahead of the crack tip for all the post-cured samples were studied using SEM. *JEOL JSM-IT300* machine operated at 20 keV was used to examine the surface morphology of the composite samples. Initially, the composites samples of size 10 mm × 10 mm were preparation. Subsequently, the samples were coated with gold using a sputter coating machine to avoid a built-up surface charge.

**Figure 12:**
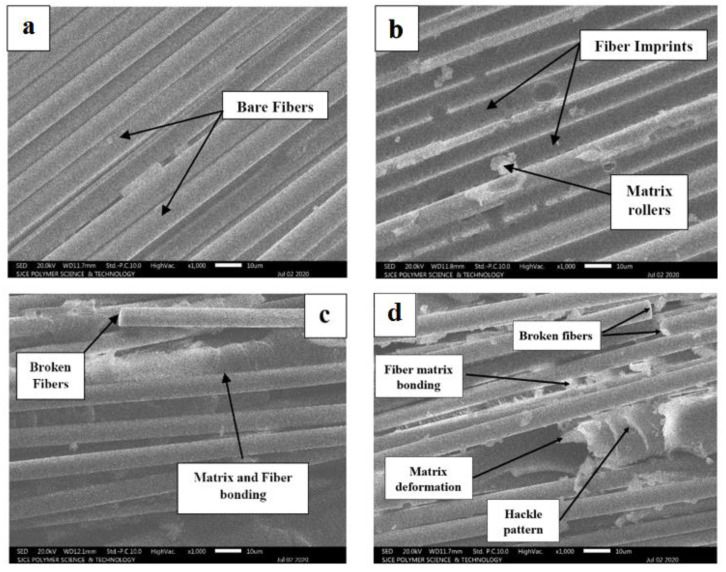
SEM micrographs from mode-I fractured delaminated surfaces of (**a**) GE-RT (**b**) GE-90-6 (**c**) GE-135-6 (**d**) GE-180-6.

**Figure 13:**
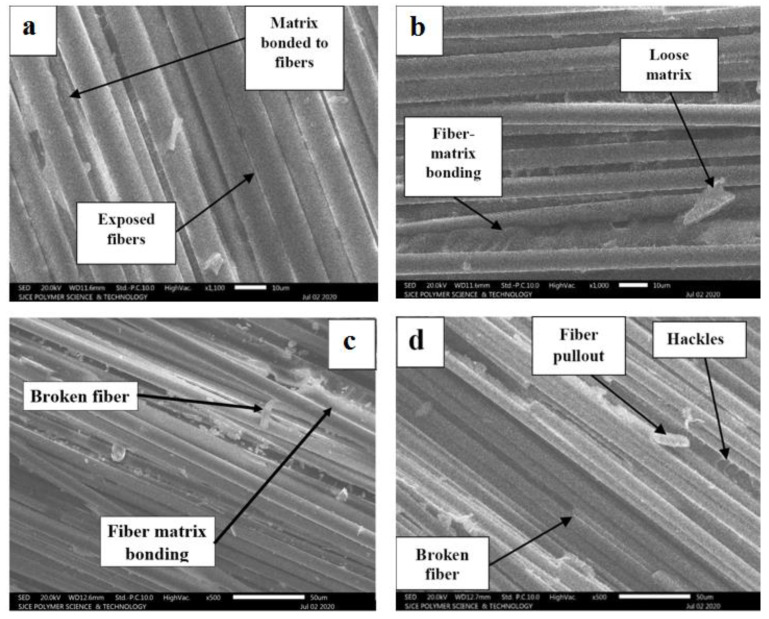
SEM micrographs from mode-I fractured delaminated surfaces of (**a**) GCE-RT (**b**) GCE-90-6 (**c**) GCE-135-6 (**d**) GCE-180 -6.

SEM micrographs of fracture surfaces of GE and GCE laminates are shown in Figure 12 and Figure 13 respectively. SEM images are studied based on matrix–fiber interfacial bonding, matrix deformation, fiber pullout, fiber breaks, and fiber bridging [30,31,32,33]. For the GE-RT composite laminate, the fibers are clearly visible with no matrix adhesion indicating a weak fiber–matrix interfacial bonding (see Figure 12a). From Figure 12b, the imprints of the fiber can be seen without significant deformation of the matrix which implies a brittle fracture. From Figure 12b,c, GE-135-6 laminates exhibit better interfacial bonding compared to GE-90-6 laminates. Therefore, GE-135-6 shows better resistance to delamination as compared to GE-90-6. The micrograph of GE-180-6 shown in Figure 12d is characterized by the hackle pattern and extended parts of the matrix which indicate plastic deformation of the matrix, implying a good fiber–matrix interfacial bonding. Broken fibers can also be observed, indicating the effect of fiber bridging.

As the post-curing temperature increases, an increase in resin ductility is observed characterized by plastic deformation in the matrix which leads to increased delamination resistance. For the GCE laminates post-cured using a constant temperature, from Figure 13b–d GCE-180-6 laminates exhibit relatively better fiber–matrix bonding characterized by the hackle pattern indicating plastic deformation of a matrix concerning GCE-RT, GCE-90-6, and GCE-135-6. As a result, GCE-180-6 demonstrates higher delamination resistance as compared to other GCE laminates. The post-curing temperature was found to have a substantial effect on fiber–matrix interfacial bonding. The post curing process promotes crosslinking that leads to the creation of long chains and cross-linked polymeric structures in the matrix. In cured composites, the matrix is relatively infiltrated between the fibers. The matrix seems effectively bonded to the fibers in composites post-cured at high temperatures [33,34]. Therefore, during the delamination of post-cured laminates under opening mode, the matrix undergoes deformation. The fiber–matrix interface resists the delamination and matrix undergoes deformation during the process, which is more prevalent in the laminates cured at higher temperature, due to a better fiber–matrix bonding.

Overall, from Figure 12 and Figure 13, it is evident that GCE laminates display better fiber–matrix adhesion, matrix plastic deformation, and fiber bridging as compared to GE laminates. The increase in the delamination resistance can be attributed to the increase in resin ductility and fiber–matrix interfacial bond with an increase in post-cure temperature.

## 6. Conclusions

The effect of process-induced residual stresses on mode-I interlaminar fracture toughness of GE and GCE composite laminates was investigated. The load drop in GE and GCE *P-δ* plots was not significant. The crack growth in GE (except GE RT) and GCE laminates was stable and no ‘stick-slip’ behavior was observed in *P-δ* plots. The loads required for the commencement of delamination in hybrid GCE laminates were higher compared to GE composite laminates. Post-cured GE and GCE laminates displayed higher displacement in DCB arms compared to RT-cured laminates for the delamination of the same crack length. The composite laminates post-cured at 180 °C for 6 h exhibited higher *G_I_* in their corresponding composite configurations.

For the GE and GCE composites, as the post-curing temperature increased, the transverse residual stresses also increased and a gradual increase in *G_I_* was also observed. The increase in the *G_I_* of GE and GCE laminates compared to corresponding laminates cured at RT can directly be related to a gradual increase in compressive residual stresses. As the crack propagates, compressive residual stresses contribute to the energy release rate in the form of additional laminate arms displacement. From the SEM micrographs, the increase in *G_I_* of GE and GCE composites can also be attributed to the increase in matrix deformation and an improved fiber–matrix interfacial bonding with an increase in post-curing temperature.

## Figures and Tables

**Table 1:** Laminate Codes for GE and GCE laminates based on post-curing conditions.

Laminate Code		Post-Curing Temperature (°C)	Curing Time (h) (min)	Cooling Rate (°C/min)
GE laminates	GCE Laminates			
GE-RT	GCE-RT	Room temperature	-	-
GE-90-6	GCE-90-6	90	6 (360 min)	20
GE-135-6	GCE-135-6	135	6 (360 min)	20
GE-180-6	GCE-180-6	180	6 (360 min)	20

**Table 2:** Dimensions of slitting specimen.

Length (mm)	Width (mm)	Thickness (mm)	
		GE Laminates	GCE Laminates
**75**	20	3.0	2.4

**Table 3:** Overall transverse residual stresses in GE and GCE laminates.

GE Laminates	Transverse Residual Stresses (MPa)	GCE Laminates	Transverse Residual Stresses (MPa)
GE-RT	−0.50	GCE-RT	−1.07
GE-90-6	−1.27	GCE -90-6	−2.07
GE-135-6	−1.62	GCE -135-6	−2.89
GE-180-6	−1.98	GCE -180-6	−4.37

## Data Availability

Not applicable.

## Supplementary Materials

The following supporting information can be downloaded at: https://www.mdpi.com/article/10.3390/ma15207135/s1, Table S1: Elastic constants of Carbon/Epoxy (CE) composite laminates; Table S2: Elastic constants of Glass/Epoxy (GE) composite laminates; Table S3: CTE of Epoxy, Carbon and Glass fibers.
