# Supplementary material for: On the Residual Stresses and Fracture Toughness of Glass/Carbon Epoxy Composites
Source: Materials (Basel). 2022 Oct 13;15(20):7135. doi: 10.3390/ma15207135 (PMC9609049; doi:10.3390/ma15207135)
Supplement: Supplementary file 1 [file materials-15-07135-s001.zip › materials-1940404-supplementary.pdf]

Table S1. Elastic constants of Carbon/Epoxy (CE) composite laminates

Details of post-curing	CE laminates	E_{11} (GPa)	E_{22} (GPa)	G_{12} (GPa)	ν_{12}
Cured under room temperature (RT)	CE-RT	55.66	2.60	0.89	0.36
Samples were held at 90°C for 6 hours and cooled to RT using a cooling rate of 20°C /min.	CE-90-6	51.69	3.12	0.77	0.33
Samples were held at 135°C for 6 hours and cooled to RT using a cooling rate of 20°C /min.	CE -135-6	49.83	2.40	0.74	0.31
Samples were held at 180°C for 6 hours and cooled to RT using a cooling rate of 20°C /min.	CE -180-6	46.96	2.08	0.73	0.30

Table S2. Elastic constants of Glass/Epoxy (GE) composite laminates

Details of post-curing	GE laminates	E_{11} (GPa)	E_{22} (GPa)	G_{12} (GPa)	ν_{12}
Cured under room temperature (RT)	GE-RT	5.375	2.08	0.96051	0.21
Samples were held at 90°C for 6 hours and cooled to RT using a cooling rate of 20°C /min.	GE-90-6	5.70049	2.168	0.99421	0.225
Samples were held at 135°C for 6 hours and cooled to RT using a cooling rate of 20°C /min.	GE -135-6	6.15629	2.274	1.03662	0.235
Samples were held at 180°C for 6 hours and cooled to RT using a cooling rate of 20°C /min.	GE -180-6	5.98268	2.448	1.06536	0.24

Table S3. CTE of Epoxy, Carbon and Glass fibers

Material	CTE along longitudinal direction (0º) $\left(\frac{m}{m°C}\right)$	CTE along transverse direction (90º) $\left(\frac{m}{m°C}\right)$
Epoxy	60×10^{-6}	60×10^{-6}
Carbon fiber	-1×10^{-6}	26×10^{-6}
Glass fiber	4.9×10^{-6}	4.9×10^{-6}
